# Clinical study on combined “lanzhou prescription” for the treatment of “syndrome of heat-toxin congestion and excessiveness” in children with acute aplastic anemias

**DOI:** 10.4314/ahs.v23i2.81

**Published:** 2023-06

**Authors:** Niancheng Yu, Hong Huang, Fang Wu

**Affiliations:** 1 Department of Traditional Chinese Medicine, Children's Hospital Affiliated to Medical College of Zhejiang University, Hangzhou 310057, Zhejiang Province, China; 2 School of Basic Medical Sciences, Zhejiang Chinese Medical University, Zhejiang Hangzhou, 310053, China

**Keywords:** Acute aplastic anemia in children, Lanzhou prescription, Traditional Chinese medicine combined with Western medicine, Clinical research

## Abstract

**Purpose:**

The clinical efficacy of “Lanzhou prescription” plus or minus combined with western medicine in the treatment of children with acute aplastic anemia, ‘excessive accumulation of heat toxin’, was comprehensively and objectively evaluated.

**Methods:**

Sixty children diagnosed with acute aplastic anemia, ‘excessive accumulation of heat toxin’ were divided into observation group (lanzhou prescription plus or minus combined with immunosuppressive therapy) and control group (immunosuppressive therapy alone). The relief degree of clinical symptoms and signs and the change of laboratory indicators were taken as the evaluation criteria.

**Results:**

(1) After treatment, the results of remission rate of two groups treated by western medicine shows that the remission rate of the observation group was significantly higher than the control group (P< 0.05). (2) the ‘cure rate’ of the observation group treated for 6 months was significantly higher than treated for 3 months (P<0.05). (3) After treated for 6 months, the indexes of CD34+ cells and FOXP3+ regulatory cells in bone marrow of observation group increased, while the CD8+ cells and B cells decreased significantly, and the indexes of CD3+ cells, CD4+ cells and NK cells decreased somewhat(P<0.05).

**Conclusion:**

Compared with immunosuppressive therapy, lanzhou prescription plus or minus combined with immunosuppressive therapy can alleviate the clinical symptoms and signs of children more effectively, obviously improve the Traditional Chinese Medicine symptoms of children, and help bone marrow hematopoietic stem cells gradually restore hematopoietic function.

## Introduction

Acute aplastic anemia is a bone marrow hematopoiesis dysfunction disease characterized by hematopoietic stem cell injury and peripheral blood pancytopenia. It is one of the most common and serious blood diseases in pediatrics, which belongs to the category of “acute marrow fatigue” in traditional Chinese medicine, and at the beginning of clinical practice, shows the manifestation of “heat-toxin congestion and excessiveness”. Its common symptoms are anemia and thrombocytopenia, and the initial symptoms are not obvious. Usually, there is no enlargement of liver, spleen and lymph nodes (except infection). Clinically, it is characterized by blood transfusion dependence, low cure rate, more adverse reactions of drug treatment, and low quality of life of patients. Traditional Chinese medicine will be aplastic anemia definition is pulp, acute aplastic is urgent pulp, chronic aplastic is slow pulp. Many doctors through theoretical and clinical practice exploration, put forward the theory of traditional Chinese medicine treatment of myeloid anemia, promote the traditional Chinese medicine in the treatment of aplastic anemia has an outstanding role, the combination of traditional Chinese and western medicine treatment of myeloid fatigue has achieved better curative effect at the same time can reduce the occurrence of western medicine adverse reactions. At present, the western medicine treatment of acute aplastic anemia in children mainly includes bone marrow transplantation, immunosuppressant (ATG or CsA) and oral administration of traditional drugs (androgen). However, bone marrow transplantation is affected by many factors, such as difficult donor source, high clinical risk, expensive treatment price, etc., so its application scope in China is limited.

Moreover, traditional medicines can only play a supporting role. The effective rate of immunosuppressive therapy is 60%-80%, with not ideal curative effect. And big side effects of western-medicine clinical medication and not ideal curative effect is an urgent problem to be solved in the treatment of acute aplastic anemia in children. “Lanzhou Prescription” was originally an effective prescription for the treatment of leukemia, aiming to “strengthen and consolidate the healthy energy, with a definite curative effect clinically. At present, at the recommended dosage, no other side effects have been found in Lanzhou Prescription except liver and kidney damage, which is a very common side effect in traditional Chinese medicine and inevitable. Considering that the basic pathogenesis of “syndrome of heat-toxin congestion and excessiveness” in children with acute aplastic anemia is “deficiency of qi and blood, insufficiency of positive qi, and invasion of heat toxin”, our hospital adopts associated “Lanzhou Prescription” and heat-clearing detoxifying Chinese herbal medicines for the treatment of acute aplastic anemia. After practice, it is found that such therapy has good clinical efficacy.

In this paper, the therapy of associated “Lanzhou Prescription” and immunosuppressant was used for the treatment of “syndrome of heat-toxin congestion and excessiveness” in children with acute aplastic anemia , and its symptoms, signs and laboratory indicators were observed and evaluated, which obtains the satisfactory curative effect, and provides a clinical basis for the integrative Chinese and western medicine therapy of “syndrome of heat-toxin congestion and excessiveness” in children with acute aplastic anemias.

## Clinical data

Sixty children with acute aplastic anemia were enrolled in this study from the hematology Department of Children's Hospital Affiliated to Zhejiang University School of Medicine from January 2009 to June 2015, including 32 males and 28 females, aging from 2-18 years old. The diagnostic criteria were based on the Diagnostic Criteria of Aplastic anemia in China revised by Chinese Medical Association in 1987 1 and the Guidelines for Clinical Research of New Chinese Medicine issued by Ministry of Health of the People's Republic of China in 1993. And the patients were divided into the observation group (n = 30) and control group (n = 30) by simple randomization. During the observation, due to severe infection death and loss to follow-up, 25 cases in the observation group and 27 cases in the control group were eventually included in the experiment.

### Therapies

**Observation group:** On the basis of immunosuppressant ATG and immunosuppressant CsA therapies, associated “Lanzhou prescription” and (12g of dried radix rehmanniae, 12g of Yam, 9g of cornus officinalis, 6g of cortex danpi, 12g of Poria cocus, 9g of Alisma rhizoma, 12g of Radix Pseudostellariae, 9g of radix ophiopogon, 6g of cassia twigs, 9g of paeonia lactiflora, 6g of ginger, 12g of Jujube, 6g of Zhi Licorice and 15g of wheat) were added. Additionally, for patients with heat-toxic exchange, 12g of Sculellaria barbata, 12g of hedyotis diffusa, 9g of prunella subtilis, 12g of polygonum cuspidatum and 12g of Paris Polyphylla Smith were added separately. For patients with blood-heat bleeding, 30g of buffalo horn or 3g of antelope horn was added, and for patients with heat congestion, 3g of safflower, 9g of salvia miltiorrhiza and 3g of hirudo were added orally.

**Control group:** 3.75mg/kg/d of rabbit anti-human thymocyte immunoglobulin (ATG) was intravenously injected for 5 days. 6mg/kg/d of cyclocytosinin A (CsA) was intravenously injected for 6 months. Then the dose was adjusted according to the blood concentration of 200-400ng/L, and supportive therapies were given according to the condition, with the treatment course of 6 months.

### Observation methods

Patients in the observation group and control group were both followed up for 6 months, with once every 3 months. Then the immediate curative effect was evaluated at the end of 3 months and 6 months of treatment course respectively.

### Statistical analysis methods

All data were statistically analysed and processed by SPSS17.0 software. The qualitative indicators and quantitative indicators are respectively described by frequency table, percentage or composition ratio, and mean ± standard deviation. Moreover, chi-square test was used for counting data, T test was used for comparison of means of measurement data between the two groups, one-way variance analysis was used for comparison of means between multiple groups, and P<0.05 was considered that the differences have statistically significance.

## Results

Comparison of “remission rate” of western-medicine efficacy evaluation between the two groups after six months of treatment course. (See Table 1 and Table 2)

**Table 1:** Comparison of western medicine efficacy evaluation between the two groups after three and six months of treatment course

Group	Treatment course	Basic cure	Remission	Obvious improvement	Improvement	Inefficient	Total	Efficacy rate	Remission rate
Therapy group	Three months	0	0	0	16	10	26	61.54%	-
Therapy group	Six months	0	13	2	6	4	25	84.00%	52.00%
Control group	Three months	0	0	0	15	13	28	53.57%	-
Control group	Six months	0	3	5	12	7	27	74.07%	11.11%

**Table 2:** Comparison of remission rates of western medicine efficacy evaluation between the two groups after three and six months of treatment course

Group	In remission (%)	Not in remission (%)	Total	χ^2^	*P*
Observation group	13 (52.00)	12(48.00)	25	10.188	0.001
Control group	3 (11.11)	24(88.89)	27		

**Note:** After the two groups of data were tested by x2, P=0.001, P<0.05, indicating that there was statistically significant difference in remission rate of western medicine efficacy evaluation after 6 months of treatment course

According to the comparison of “remission rate” between the two groups after six months of treatment course, it is found that the remission rate in the observation group was significantly higher than in the control group, and the difference was statistically significant (P<0.05), confirming that associated “Lanzhou Prescription” and immunosuppressive therapy was significantly superior to immunosuppressive therapy alone in remitting western-medicine clinical symptoms of children.

Comparison of “cure rate” of traditional Chinese medicine efficacy evaluation between the two groups after three and six months of treatment course. (See Table 3 and 4)

**Table 3:** Comparison of traditional Chinese medicine efficacy evaluation in the observation group after3 and 6 months of treatment course

Treatment course	Cure	Excellent	Efficient	Inefficient	Total	Efficacy rate	Cure rate
Three months	1	6	15	4	26	84.62%	3.85%
Six months	6	11	6	2	25	92.00%	24.00%

**Table 4:** Comparison of cure rate of traditional Chinese medicine efficacy evaluation in the observation group after3 and 6 months of treatment course

Treatment course	In cure (%)	Not in cure (%)	Total	χ^2^	*P*
Three months	1 (3.85)	25(96.15)	26	4.372	0.037
Six months	6 (24.00)	19(76.00)	25		

**Note:** After the two groups of data were tested by x2, P=0.037, P<0.05, indicating that there was statistically significant difference in the cure rate of traditional Chinese medicine efficacy evaluation after3 and 6 months of treatment course.

According to the comparison of “cure rate” of traditional Chinese medicine efficacy evaluation after 3 months of treatment course and 6 months of treatment course, it can be seen that the efficacy after 6 months of treatment course was significantly higher than that after 3 months of treatment course, and the difference was statistically significant (P<0.05), confirming that in the observation group, the efficacy after 6 months of treatment course was significantly superior to that after 3 months of treatment course in curing traditional Chinese medicine symptoms of children.

Comparison of bone marrow immunology indicators in the observation group before treatment and after 6 months of treatment course (See Table 5)

**Table 5:** Comparison of bone marrow immunology indicators in the observation group before treatment and 6 months after treatment

Immunological indicators	Before treatment	6 months after treatment	t	*P*
CD34+	0.28±0.16	0.89±0.46	4.820	0.000
FOXP3+	0.21±0.21	2.85±2.55	3.297	0.005
CD3+	57.86±9.21	40.48±20.12	2.965	0.007
CD4+	22.97±9.78	14.12±9.02	2.355	0.027
CD8+	27.80±5.72	18.36±7.80	3.567	0.002
B	13.67±5.19	6.81±3.77	3.717	0.001
NK	13.55±8.81	7.46±5.10	2.122	0.044

According to the comparison of efficacy before treatment and after 6 months of treatment course, it is found that in the observation group, indicators of CD34+ cells and FOXP3+ regulatory cells in bone marrow were increased, indicators of CD8+ cells and B cells were decreased significantly, indicators of CD3+ cells, CD4+ cells and NK cells were decreased, and the differences were statistically significant (P<0.05), proving that the combination of “Lanzhou Prescription” and immunosuppressive therapy can restore the function of T cells, so as to effectively restore the hematopoietic function of bone-marrow hematopoietic stem cells.

## Discussion

Modern medical studies have shown that aplastic anemia can be caused by the disproportion of bone marrow T cell subsets, early activation of T cells, increased secretion of hematopoietic negative regulatory factors, promotion of hematopoietic progenitor cell apoptosis, clonal proliferation of T cells, abnormal immunity and other mechanisms 2-7. And for the treatment of aplastic anemia, clinical applications of immunosuppression and stem cell transplantation can improve or cure the symptoms of about 60% of patients 8-9. At present, it has been basically agreed at home and abroad that most aplastic patients present abnormal activation of T lymphocytes, which leads to inhibitory cytokines are increased abnormally or activated T cells directly act on hematopoietic stem/progenitor cells, thus resulting in bone marrow dysfunction 10. Strengthening immune suppression therapy, there are many side effects, like ATG treatment, because it belongs to the biological agents, at the beginning of application, there may be some immune reaction, or an allergic reaction, so we definitely need to take some allergy before using, especially the application of the adrenal glucocorticoid, it will take us at least two weeks. Then pay attention to the serum disease response, ATG after the use of serum disease reaction, generally occurs after treatment, which should take active preventive measures. At the same time, there is a process of inhibition on blood image. At the beginning, there is a period of decline. At this time, we need to strengthen a lot of support, including the infusion of blood products, red blood cells, platelets, especially platelets. Sometimes one dose is not enough, or even two or three doss a day, because without strong support, the previous treatment would be wasted. Before the ATG can work, the patient may suddenly die of bleeding, or infection, so the prophylactic use of antibiotics is a must.

The Annual Meeting and Proceedings of the American Society of Hematology in 2000--2006 and the Guidelines for the Diagnosis and Treatment of aplastic anemia issued by the British Standards Committee of Hematology indicated that at present, immunosuppressants and bone marrow transplantation are the main clinical therapies for aplastic anemia, and androgen is rarely adopted, only as a “remedial” or “alternative” treatment 11-12. The big side effect of western-medicine clinical medication and not ideal curative effect is an urgent problem to be solved in the treatment of aplastic anemia. While traditional Chinese medicine has definite curative effect, small toxic and side effects, low cost and wide clinical application. Therefore, the combination of traditional Chinese medicine and immunosuppressants in the treatment of children with acute aplastic anemia, is of more and more bright clinical application prospects.

In traditional Chinese medicine, the disease name of “acute aplastic anemia” is not existent. According to the symptoms of “acute aplia anemia” such as sudden onset, and its characteristics such as pallor, weakness of body, palpitation, shortness of breath, high fluctuation of pulse, as well as accompanied severe bleeding, infection and high fever, “acute aplastic anemia” is mostly classified into the category of “acute fatigue”, “hot fatigue”, “blood syndrome” or “febrile disease” in the treatises of physicians of past dynasties 13. In Synopsis of Golden Chamber compiled by Zhang Zhongjing, the name of “deficiency fatigue” was first put forward. According to Chao Yuan fang's general treatise on the Cause and Symptoms of Diseases, it can be seen that the “deficiency fatigue and heat excessiveness” mentioned was similar to the clinical symptoms of non-infectious fever of modern “aplastic anemia”. And Yu Jiayan's Medical Laws first proposed three clinical symptoms similar to aplastic anemia, namely anemia, bleeding and infection 14.

Although acute aplastic anemia can be generally called “deficiency fatigue” due to the bone marrow is damaged and there is no bleeding in marrow, it is better to be called as “marrow fatigue” to directly reflect the essence of the disease. Therefore, in 2007, Wuhan conference attributes “aplastic anemia” to the category of “marrow fatigue diseases” in traditional Chinese medicine. Similarly, “acute aplastic anemia” is “acute marrow fatigue disease”. Children are in the stage of growth and development, and the compensatory function of their bone marrow is far less than that of adults, so the incidence of “acute marrow fatigue disease” in aplastic anemia children is higher. According to the report of Blood Research Institute of Chinese Academy of Sciences, before the implementation of hematopoietic stem cell transplantation and effective immunosuppressive therapy, the average survival time of acute aplastic anemia was only 3 months, and the fatality rate within half a year was as high as 90% 15, indicating a particularly high severity of acute aplastic anemia in children. However, the clinical research, concern extent and research investment in the field of pediatric hematopathy in China are insufficient, with a big gap internationally.

The basic pathogenesis of “syndrome of heat-toxin congestion and excessiveness” in children with acute aplastic anemia is “deficiency of qi and blood, insufficiency of positive qi, and invasion of heat toxin”, namely, “deficiency in essence and asthenia in superficiality”, so “toxification and purgation in combination” should be taken as its treatment principle 16. The combined “Lanzhou Prescription” is consisted of key liuwei rehmannia decocation, pulse-activating decocation and cassia twig decocation, as well as heat-clearing and detoxifying Chinese herbs, which has the functions of strengthening and consolidating the healthy energy, tonifying the kidney and spleen, clearing heat, detoxifying, cooling blood and stopping bleeding. Among them, its traditional Chinese medicine components have the functions of regulating immune and improving microcirculation. In this regard, “Lanzhou Prescription” can regulate T cell function recovery, and gradually restore hematopoietic function of bone-marrow hematopoietic stem cells, so as to effectively relieve the symptoms and signs in clinic, and improve the symptoms of traditional Chinese medicine in children with acute aplastic anemia. In this experiment, the combined therapy of traditional Chinese medicine and western medicine was used to treat acute aplastic anemia, which has a good curative effect.
